# Diazoxide and Exercise Enhance Muscle Contraction during Obesity by Decreasing ROS Levels, Lipid Peroxidation, and Improving Glutathione Redox Status

**DOI:** 10.3390/antiox9121232

**Published:** 2020-12-04

**Authors:** Mariana Gómez-Barroso, Koré M. Moreno-Calderón, Elizabeth Sánchez-Duarte, Christian Cortés-Rojo, Alfredo Saavedra-Molina, Alain R. Rodríguez-Orozco, Rocío Montoya-Pérez

**Affiliations:** 1 Instituto de Investigaciones Químico-Biológicas, Universidad Michoacana de San Nicolás de Hidalgo, Francisco J. Múgica S/N, Col. Felicitas del Río, Morelia, Michoacán 58030, Mexico; 0939531k@umich.mx (M.G.-B.); 0935000j@umich.mx (K.M.M.-C.); christian.cortes@umich.mx (C.C.-R.); saavedra@umich.mx (A.S.-M.); 2 Departamento de Ciencias Aplicadas al Trabajo, Universidad de Guanajuato Campus León, Eugenio Garza Sada 572, Lomas del Campestre Sección 2, León, Guanajuato 37150, Mexico; elizabeth.sanchez@ugto.mx; 3 Facultad de Ciencias Médicas y Biológicas “Dr. Ignacio Chávez”, Universidad Michoacana de San Nicolás de Hidalgo Av. Dr. Rafael Carrillo S/N, Esq. Dr. Salvador González Herrejón, Bosque Cuauhtémoc, Morelia, Michoacán 58020, Mexico; alain.rodriguez@umich.mx

**Keywords:** skeletal muscle, obesity, fatigue, oxidative stress, diazoxide, exercise

## Abstract

Obesity causes insulin resistance and hyperinsulinemia which causes skeletal muscle dysfunction resulting in a decrease in contraction force and a reduced capacity to avoid fatigue, which overall, causes an increase in oxidative stress. K_ATP_ channel openers such as diazoxide and the implementation of exercise protocols have been reported to be actively involved in protecting skeletal muscle against metabolic stress; however, the effects of diazoxide and exercise on muscle contraction and oxidative stress during obesity have not been explored. This study aimed to determine the effect of diazoxide in the contraction of skeletal muscle of obese male Wistar rats (35 mg/kg), and with an exercise protocol (five weeks) and the combination from both. Results showed that the treatment with diazoxide and exercise improved muscular contraction, showing an increase in maximum tension and total tension due to decreased ROS and lipid peroxidation levels and improved glutathione redox state. Therefore, these results suggest that diazoxide and exercise improve muscle function during obesity, possibly through its effects as K_ATP_ channel openers.

## 1. Introduction

Obesity is a chronic disease of preventable multifactorial origin; it is the fifth main risk factor for human death globally. It is a pathological state that impairs skeletal muscle [1,2], characterized by a decrease in the force of contraction, a reduced capacity to withstand fatigue, and cell damage [3].

Fat accumulation alters carbohydrates and lipids metabolism, which affects the normal contractile function [4], decrease in glucose uptake, and deterioration of the insulin signaling pathway, causing insulin resistance [5]. Together, it favors the development of fatigue [6], decreased mitochondrial respiration and ATP production, as well as an increase in mitochondrial production of reactive oxygen species (ROS) [7].

Opening of the ATP-sensitive potassium channels (K_ATP_ channels) has been identified as a defense mechanism to counter muscle fatigue and metabolic stress; in the cell membrane and the inner membrane of the mitochondria [8], and play an important role as sensors of intracellular ATP and ADP ratio [9], closing when ATP levels are high and opening when ADP levels increase [10]. Several studies have shown that K_ATP_ channels play an essential role in tissue protection [11,12], where the channel becomes crucial in preventing contractile dysfunction and fiber damage caused by oxidative stress [13].

K_ATP_ channels can be activated pharmacologically, making them an important target for myoprotection [14,15]. Diazoxide is a vasodilator and an inhibitor of insulin secretion. It is considered a drug that is beneficial for some pathologies, such as obesity, since it reduces intake of food and weight gain, prevents hyperinsulinemia, improves insulin sensitivity, and improves blood glucose and lipid profile, which together counteracts mitochondrial dysfunction [16,17].

Diazoxide, reported as a selective opener for mitoK_ATP_ channels, delays fatigue in mammalian fast skeletal muscle fibers [18]. Similarly, increased post-fatigue force in the slow skeletal muscle has been observed by diazoxide and nicorandil, another K_ATP_ channel opener, [19,20] and both, protects skeletal muscle against ischemia-reperfusion injury [15].

Exercise is a non-pharmacological treatment for metabolic disorders associated with obesity. It has been reported that it increases the expression of K_ATP_ channels [13] and promotes various metabolic adaptations in skeletal muscle [21]. Exercise decreases the fat stored in the muscle which improves muscle contraction, increases insulin sensitivity which prevents hyperinsulinemia and stimulates the transport of glucose into the cell [22], and increases antioxidant defenses through a hormonal mechanism which makes the muscle more resistant to oxidative stress [23]. Together these mechanisms protect against mitochondrial dysfunction through lower ROS levels by improving respiration and promoting beta-oxidation of fatty acids [7,24].

Therefore, we hypothesized that diazoxide and exercise improve muscle function in obesity by decreasing oxidative stress. Obesity and its effect on skeletal muscle have been extensively studied, as has exercise to counteract its adverse effects, while the opening of the K_ATP_ channels has been studied as a mechanism of muscular protection. However, this is the first study that shows how diazoxide, exercise, and the combination of both, improve the contraction and function of muscle fibers in obesity by reducing oxidative stress.

## 2. Materials and Methods

### 2.1. Experimental Animals and Groups

Male Wistar rats between 300 and 350 g were used. The animals were kept in acrylic cages under bioterium conditions at room temperature for a period of 12 h of light/12 h of darkness, with free access to food and water. Animals were randomly assigned to eight groups (See Table 1).

The diets are applied for eight weeks, while the protocol of moderate-intensity exercise is applied for five weeks. Diazoxide was administered for 14 days intraperitoneally at a dose of 35 mg/kg. Finally, the respective combinations were combined.

Diets were administered for eight weeks; the standard rodent chow^®^ diet showed a caloric content of 336 cal/100 g with a proportion of 28.507% protein, 13.496% fat, and 57.996% carbohydrates. The high-fat diet contained 50% standard rodent chow^®^ and 50% fat [2], showed a caloric content of 649.25 cal/100 g with a proportion of 14.05% protein, 69.5% fat, and 21.4% of carbohydrates (Mexican equivalent food system). The exercise protocol was applied for five weeks at moderate intensity (see Table 2) and diazoxide was applied at a dose of 35 mg/kg intraperitoneally for 14 days in the obese group, diazoxide was applied at the end of the obesity induction period, while in those subjected to the exercise protocol, the drug was applied during weeks four and five.

All procedures with animals were carried out by the Federal Regulations for the Use and Care of Animals (NOM-062-ZOO-1999) issued by the Ministry of Agriculture of Mexico.

### 2.2. Muscles Dissection

At the end of the protocols, the rats fasted for 12 h, the weight and glucose parameters of each group were measured to compare these values concerning each treatment. Subsequently, they were sacrificed for cervical dislocation; dissection was performed to obtain soleus and digitorum extensor longus (EDL) muscles of the two posterior extremities. The muscles of one of the extremities were maintained with Krebs-Ringer solution, to be later taken to isometric tension measurements. Simultaneously, the other extremity muscles were stored at −80 °C to be subsequently homogenized for biochemical tests. The Biuret method obtained the protein concentration of the homogenates [25].

### 2.3. Isometric Tension Measurements

The soleus and EDL muscles were placed in a Petri dish covered with a transparent resin bottom (Sylgard, World Precision Instruments, Sarasota, FL. USA) where they were fixed with the help of entomological pins immersed in Krebs-Ringer solution (118 mM NaCl, 4.75 mM KCl, 1.18 mM MgSO_4_, 24.8 mM NaHCO_3_, 1.18 mM KH_2_PO_4_, 10 mM glucose, and 2.54 mM CaCl_2_) and carbogen gas (95% O_2_ and 5% CO_2_) was supplied. Excess connective and fatty tissue were removed under a stereoscopic microscope.

The muscle was mounted into a chamber for isometric tension measurements, with its proximal end attached to the bottom of the chamber and the distal end to the hook of an optical transducer, which was connected to an amplifier and this in turn to an analog–digital interface (World Precision Instruments, Sarasota, FL. USA) that allowed acquiring the tension generated by the muscle in a computer, using the MDAC software (World precision instruments, Sarasota, FL. USA). Two platinum electrodes were placed inside the recording chamber, which was connected to a stimulus isolation unit and an electric stimulator (Grass) in order to apply the protocol to induce fatigue, which consisted of 100 V pulses, 300 ms of duration, and a frequency of 45 Hz for soleus muscle and 50 Hz for EDL muscle. The stimulation was stopped once fatigue was presented. The fatigued muscles were stored at −80 °C to be subsequently homogenized for biochemical tests’ performance (measurement of total reactive oxygen species, lipid peroxidation, and glutathione levels).

The muscle was stretched 1.3 times its resting length and left to perfuse in the physiological solution for 10 min before recording the isometric tension; the experiment was performed at a temperature of 25 °C.

### 2.4. Reactive Oxygen Species Analysis

ROS levels were determined by evaluating the oxidation of the 2′, 7′-dichlorodihydrofluorescein diacetate fluorescent probe (H_2_DCFDA). A total of 0.5 mg/mL of homogenate from each muscle was placed in test tubes and incubated at 4 °C with constant shaking for 20 min in a buffer with 10 mM HEPES, 100 mM KCl, 3 mM MgCl_2_, 3 mM KH_2_PO_4_ (pH 7.4), and 1.25 mM of H_2_DCFDA in a total volume of 2 mL. This suspension was placed in a quartz cell, and the basal fluorescence was determined. One minute later, 10 mM glutamate/malate was added as substrate, and the changes in fluorescence were determined for an additional 20 min [26].

Fluorescence changes were measured on a Shimadzu RF-5301PC spectrofluorometer (λex 485 nm; λem 520 nm). The data were expressed as the difference in fluorescence obtained by subtracting the fluorescence units obtained at the end of the 20 min with the substrate minus the fluorescence obtained before the substrate’s addition. The result was expressed as arbitrary fluorescence units/min.

### 2.5. Lipid Peroxidation Measurement

Lipid peroxidation was assessed using the levels of thiobarbituric acid reactive substances (TBARS). First, 0.5 mg/mL of the homogenate was resuspended in 1 mL of phosphate buffer (50 mM KH_2_PO_4_, pH 7.6) and incubated with 50 µM FeSO_4_ for 30 min at 4 °C to induce lipid peroxidation [27].

At the end of the incubation time, 2 mL of acid solution (trichloroacetic acid, thiobarbituric acid, and hydrochloric acid) were added to each sample, and they were incubated in boiling water for 30 min. Subsequently, the tubes were placed on ice for 5 min and centrifuged at 7500 rpm for 5 min. The absorbance of each sample was determined at 532 nm on a Shimadzu UV-2550 spectrophotometer. Data were expressed as TBARS/mg protein numbers.

### 2.6. Glutathione Redox State Measurement

To 0.5 mg/mL of muscle homogenate, 5% (*v*/*v*) sulfosalicylic acid was added, and it underwent two cycles of freezing and thawing. Subsequently, it was centrifuged at 10,000 rpm for 5 min and the supernatant was extracted.

Total glutathione (GSH + GSSG) and oxidized glutathione (GSSG) were determined by an enzymatic method. GSH + GSSG levels were determined using 90 µL of the supernatant, resuspended in phosphate buffer (K_2_HPO_4_, 0.1 M, pH 7.5), and mixed with 3 mM 5,5′-dithiobis 2-nitrobenzoic acid (DTNB) and 0.115 µ/mL glutathione reductase in a final volume of 1 m. After 5 min incubation at room temperature, 2 mM NADPH was added and the reaction kinetics were determined for 15 min. The increase in absorbance at 412 nm measured on a Shimadzu UV-2550 spectrophotometer was converted to GSH concentration using a standard curve with known GSH values [28,29].

For GSSG determination, the same DNTB recycling assay was applied after incubating for 1 h at room temperature with 4% vinylpyridine (*v*/*v*) to derivatize the reduced GSH [30]. Reduced glutathione (GSH) was determined by subtracting the concentration of GSH + GSSG minus that of GSSG. Data were expressed as µMoles/mg protein.

### 2.7. Data Analysis

Results were expressed as the mean ± standard error of n = 8 independent experiments using samples from different animals. Statistical differences between groups were determined by one-way and two-way analysis of variance (ANOVA) and Tukey’s post-hoc test. A *p* ≤ 0.05 was established. The analysis was performed with GraphPad Prism software version 6.0.

## 3. Results

### 3.1. Effect of Diazoxide and Exercise on the Physiological Parameters of Obese Rats

The effect of diazoxide, exercise, and their combination on bodyweight and fasting serum glucose levels was evaluated at the end of each treatment. Table 3 shows these values. The obese group increased bodyweight by 70.6% compared to the control group; however, in the groups of obese rats treated with diazoxide, exercise, and the combination of both, a reduction in bodyweight of 18.35% was observed, 17.62% and 22.69%, respectively, to the group of obese rats without treatments. In blood glucose levels, it was observed how obesity increased blood glucose levels by 28.08% concerning the control group; however, a reduction in said levels of 13.95% was observed in the group of obese rats treated with diazoxide, 21.49% in the exercise group, and 26.12% in the exercise with diazoxide group.

### 3.2. Effect of Diazoxide and Exercise on Maximum and Total Tension and Time of Resistance to Fatigue of Slow and Fast Skeletal Muscle of Obese Rats

**Figure 1:**
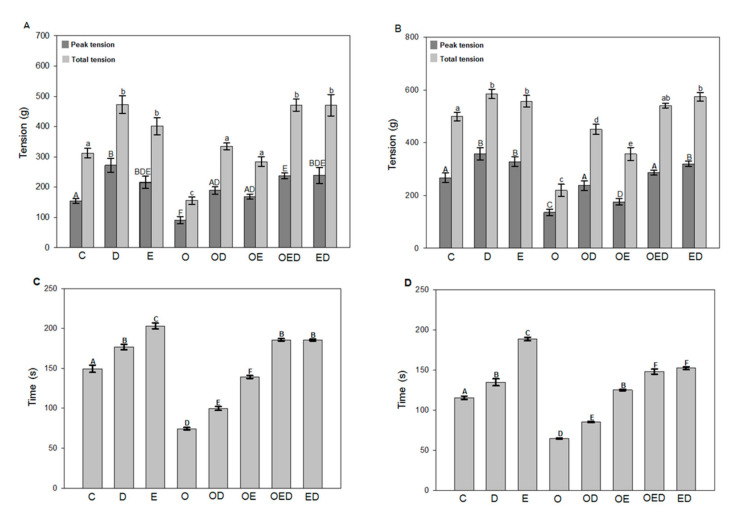
Effect of diazoxide and exercise on maximum, total tension, and time of resistance to fatigue of slow and fast skeletal muscle of obese rats. (**A**) Maximum tension and total soleus muscle tension, (**B**) maximum and total tension of the EDL muscle, (**C**) fatigue resistance time for soleus muscle, and (**D**) fatigue resistance time for EDL muscle. C: control; D: diazoxide; E: exercise; O: obese; OD: obese diazoxide; OE: obese exercise; OED: obese diazoxide exercise; ED: diazoxide exercise. Data are represented as the mean ± standard error. Different letters indicate statistically significant differences between groups, in graphs A and B, capital letters compare the maximum tension, lowercase letters compare the total tension (*p* < 0.05) two-way ANOVA, Tukey post-hoc test, *n* = 8.

To explore the effect of treatment with diazoxide, exercise, and the combination of both, maximum and total tension and fatigue resistance was assayed, a record of tension in the soleus muscle and EDL of the different groups was performed. Figure 1 shows the maximum and total tension and the resistance time to fatigue of the soleus muscle (A,C) and EDL (B,D). In the soleus muscle (Figure 1A) of obese rats, a 41.22% decrease in maximum tension, and a 50.18% decrease in total tension concerning the control group can be observed. However, an increase was observed in both tensions with each treatment, diazoxide and exercise, and even a better effect with the combination of both, since a 108.58% increment for maximum tension can be observed, and 115.03% in total tension. In comparison, the group of obese rats exercised presented an increase in maximum tension of 85.94% and 82.64% in total tension.

Finally, in obese rats exercised with diazoxide, an increase of 162.12% was observed for maximum tension and 102.77% for total tension. EDL muscle (Figure 1B) of obese rats showed a decrease of 49.15% in the maximum tension and 56.17% for the total tension. However, there was an increase of both tensions with each treatment, and a more significant increase with the combination of both, since the obese group treated with diazoxide had an increase of 74.97% for maximum tension and 105.70% in total tension. In contrast, in the group of obese rats exercised there was an increase in the maximum tension of 29.28% and 63.28% in total tension was shown.

### 3.3. Diazoxide and Exercise Decrease ROS Levels and Lipid Peroxidation in Slow and Fast Skeletal Muscle of Obese Rats

**Figure 2:**
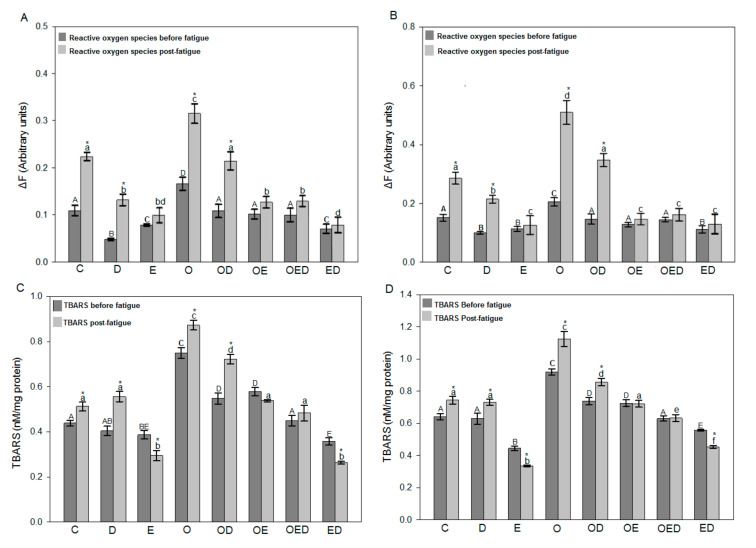
Effect of diazoxide and exercise on ROS levels and lipid peroxidation in slow and fast skeletal muscle of obese rats. (**A**) ROS levels in the soleus muscle, (**B**) ROS levels in the EDL muscle, (**C**) TBARS levels in the soleus muscle, and (**D**) TBARS levels in the EDL muscle, before and after fatigue. C: control; D: diazoxide; E: exercise; O: obese; OD: obese diazoxide; OE: obese exercise; OED, obese exercise with diazoxide; ED: exercise with diazoxide. Data are represented as the mean ± standard error. Different letters indicate statistically significant differences between the groups, capital letters compare the different groups before fatigue, and lowercase letters compare the different groups after fatigue. * indicates significant differences in the comparison of the same group before and after fatigue (*p* < 0.05) two-way ANOVA, Tukey’s post-hoc test, *n* = 8.

To analyze the effect of diazoxide treatment and the combination of both on the oxidative stress of muscle tissue, ROS levels, and lipid peroxidation of the different groups’ soleus and EDL muscles were evaluated. Figure 2 shows ROS and lipid peroxidation levels of the soleus muscle (Figure 2A,C) and EDL (Figure 2B,D) before and after fatigue. In the evaluation of ROS in the soleus muscle (Figure 2A) and the EDL muscle (Figure 2B), there was an increase in ROS levels of 51.96% and 35.89% before fatigue and 40.81% and 81.41% after fatigue, respectively, in the group of obese rats compared to the control group. However, a decrease in ROS levels with each of the treatments was observed; in the obese group treated with diazoxide, there was a decrease in ROS levels of 34.80% for soleus muscle and 45.46% for EDL muscle before fatigue, and 26.67% for soleus muscle and 29.26% after fatigue. The exercised obese group showed a decrease of 38.69% in soleus muscle and 28.59% for EDL muscle before fatigue and 58.57% in soleus muscle and 71.73% for EDL muscle after fatigue and finally in the group of obese rats exercised with diazoxide, a decrease of 39.95% for soleus muscle and 37.25% for EDL muscle before fatigue and 63.49% for soleus muscle and 69.38% for EDL muscle after fatigue, all compared to the group of obese rats without any treatment.

In lipid peroxidation, the results of the soleus muscle (Figure 2C) and EDL muscle (Figure 2D) showed an increase in lipid peroxidation levels of 70.91% before fatigue and 70.19% after fatigue and 43.48% before fatigue and 51.19% and after fatigue, respectively, in the group of obese rats compared to the control group. However, a decrease in lipid peroxidation levels was observed with each of the treatments separately and a synergistic effect with both. In the group of obese rats treated with diazoxide, a decrease of 26.91% was observed for soleus muscle and 19.70% for EDL muscle before fatigue, and 17.24% for soleus muscle and 23.91% for EDL muscle after fatigue. The group of exercised obese rats showed a decrease of 22.84% for the soleus muscle and 21.07% for the EDL muscle before fatigue, and 38.36% for the soleus muscle and 35.87% for the EDL muscle. Finally, in the group of obese rats exercised with diazoxide, a decrease of 39.96% was observed for soleus muscle and of 31.46% for EDL muscle before fatigue and 44.64% for soleus muscle and 43.77% for EDL muscle after fatigue, concerning the obese group without treatment.

### 3.4. Effect of Diazoxide and Exercise on Glutathione Redox Status in Slow and Fast Skeletal Muscle of Obese Rats

**Figure 3:**
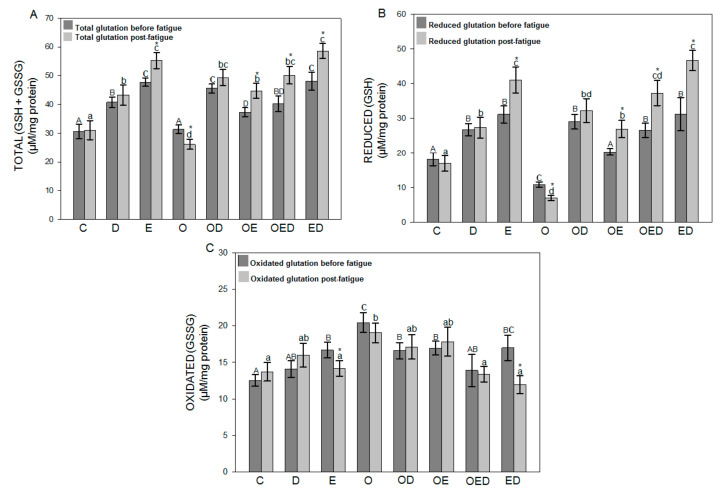
Effect of diazoxide and exercise on glutathione redox status in slow skeletal muscle of obese rats. (**A**) Total glutathione soleus muscle, (**B**) reduced soleus glutathione muscle, and (**C**) oxidized glutathione soleus muscle, before and after fatigue. C: control; D: diazoxide; E: exercise; O: obese; OD: obese diazoxide; OE: obese exercise; OED, obese exercise with diazoxide; ED: exercise with diazoxide. Data are represented as the mean ± standard error. Different letters indicate statistically significant differences between the groups, capital letters compare the different groups before fatigue, and lowercase letters compare the different groups after fatigue. * indicates significant differences in the comparison of the same group before and after fatigue (*p* < 0.05) two-way ANOVA, Tukey’s post-hoc test, *n* = 8.

**Figure 4:**
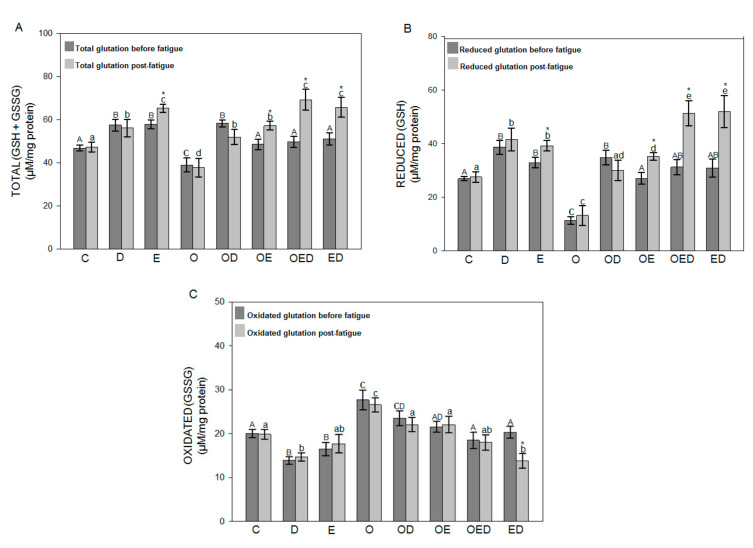
Effect of diazoxide and exercise on glutathione redox status in fast skeletal muscle of obese rats. (**A**) EDL muscle with total glutathione, (**B**) EDL muscle with reduced glutathione, and (**C**) EDL muscle with oxidized glutathione, before and after fatigue. C: control; D: diazoxide; E: exercise; O: obese; OD: obese diazoxide; OE: obese exercise; OED, obese exercise with diazoxide; ED: exercise with diazoxide. Data are represented as the mean ± standard error. Different letters indicate statistically significant differences between the groups, capital letters compare the different groups before fatigue, and lowercase letters compare the different groups after fatigue. * indicates significant differences in the comparison of the same group before and after fatigue (*p* < 0.05) two-way ANOVA, Tukey’s post-hoc test, *n* = 8.

To evaluate the effect of diazoxide, exercise, and both on glutathione’s redox status in obesity, Figure 3 and Figure 4 show the results obtained for total glutathione levels (GSH + GSSG), reduced glutathione (GSH), and oxidized glutathione (GSSG) before and after inducing fatigue for the soleus muscle and EDL muscle, respectively. These results indicate that during obesity no differences were observed in the levels of GSH + GSSG before and after fatigue in relation to the control group in the soleus muscle (Figure 3A), while in the EDL muscle (Figure 4A) a decrease in GSH + GSSG levels of 16.74% before fatigue and 20.12% after fatigue was observed in relation to the control group. However, in the group of obese rats treated with diazoxide, a 45.31% increase was observed for soleus muscle and 49.38% for EDL muscle before fatigue and 88.90% for soleus muscle and 37.48% for EDL muscle after fatigue. The group of obese rats exercised showed an increase of 18.80% before fatigue for soleus muscle and 24.37% for EDL muscle and 71.42% for soleus muscle and 51.38% for EDL after fatigue. Finally, in the group of obese rats exercised with diazoxide, an increase of 28.16% was observed for soleus muscle and 27.27% for EDL muscle before fatigue and 92.23% for soleus muscle and 83.33% for EDL muscle after fatigue in relation to the group of obese rats without treatment. Regarding the GSH levels of the soleus muscle (Figure 3B) and EDL (Figure 4B), the group of obese rats showed a decrease in levels of 39.76% for soleus muscle and 57.84% for EDL muscle before fatigue and a decrease of 58.25% for soleus muscle and 52.20% for EDL muscle after fatigue to control. However, an increase in GSH levels could be seen in obese rats with each of the treatments, concerning obese rats without treatment.

To conclude, it should be mentioned that the levels of oxidized glutathione did not show significant differences between the muscles analyzed before and after fatigue in both muscles of each group evaluated.

## 4. Discussion

More than a third of the world’s adult population suffers from obesity [30], with more than 500 million people worldwide. Obesity is related to many pathologies [7] and is exacerbated by a sedentary lifestyle and lack of physical activity (WHO, 2016) [31]. Skeletal muscle dysfunction is a complication of obesity, as it causes significant atrophy, leading to a decrease in the muscle contraction force, reduced ability to support fatigue, and numerous metabolic alterations and increased oxidative stress [1,3,6].

Studies conducted by Alemzadeh et al. [17], Pompeani et al. [1], Bae et al. [5], and Lu et al. [32] showed that obesity-induced with a high-fat diet increases bodyweight and plasma glycemia in Wistar rats. The results in Table 3 show consistency with these studies for both parameters. However, in this study, it was observed that both the treatment with diazoxide, the exercise protocol, and the combination of both decreased bodyweight and plasma glycemia during obesity, effects that may be related to the reduction in the consumption of food, improvement of basal metabolic rate, reduction of lipogenesis, improvement of insulin sensitivity and glucose transport, and suppression of hyperinsulinemia [5,17,22,33].

A higher concentration of lipids in muscle tissue produces skeletal muscle dysfunction [6]. This experimental series observed how muscle contraction and fatigue resistance were decreased in the soleus muscle and EDL of obese rats (Figure 1). The results showed that obesity affects the normal contractile function of muscles, which is associated with an increase in intramuscular fat, causing a deficiency in the muscle ability to contract [6], an increase in proteolysis, change in fiber type, metabolic alterations, and increased oxidative stress [1,7,34].

Previous studies have shown that treatment with diazoxide prevents and reverses metabolic disorders, such as loss of insulin sensitivity, and has been associated with an improvement in glucose transport and the promotion of lipid metabolism [6], which directly or indirectly improves the functioning of muscle tissue.

In this study, it was observed that the treatment with diazoxide increased muscle contraction and promoted resistance to fatigue in the obese group treated with this drug (Figure 1); data are consistent with what was observed by García et al. [18] where diazoxide increased the post-fatigue tension of the mouse EDL muscle. This suggests that the protective effect of diazoxide in obesity may be due to the opening of KATP channels, leading to an increase in cellular respiration through the electron transport chain (CTE) and an increase in the synthesis of ATP [35], favoring muscle contraction and resistance to fatigue [36,37]. A very similar effect was observed with implementing the exercise protocol during obesity (Figure 1). Studies by Zigman et al. [38] and Kraljievic et al. [13] showed an exercise-induced increase in the expression of cardiac sarcolemmal KATP channels, which improved conditioning and contraction, a process that is not ruled out could be occurring in our results, as improvement in muscle contraction and fatigue resistance. Similarly, the positive effects of exercise are attributed to the decrease in intramuscular fat, as a consequence of the increase in skeletal muscle metabolism [3], or the regulation of numerous pathways of signaling in which exercise participates by improving its functioning, such as insulin sensitivity, glucose transport, lipid profile, and reduced stress markers [3,5].

The increase in fat stored in skeletal muscle has been associated with increased ROS levels, the appearance of oxidative stress, and eventual cell damage [7].

Abrigo et al. [2] evaluated ROS levels in obese mouse muscles induced by a high-fat diet. They observed in this group an increase in ROS levels 7.22 times that of the control group. This is consistent with the obtained results in this work since it was possible to observe increased ROS levels in the groups of obese rats, compared to the control group in both muscles before and after the fatigue was induced (Figure 2A,B), observing a higher level of ROS in EDL muscle because fast-twitch fibers are more susceptible to oxidative stress than slow-twitch fibers [39]. The increase in ROS levels during obesity is due to the increase in intramuscular fat causing a reduction in the muscle’s ability to contract [6], which causes reduced ATP levels and decreases mitochondrial volume producing mitochondrial dysfunction and promoting muscle fatigue [36,37,39]. It has been shown that there is a dose-dependent relationship between ROS concentration and reduced muscle contractility and the occurrence of fatigue [40]. This work showed that the use of diazoxide, regular exercise, and the combination of both reduced ROS levels in the group of obese rats in both soleus muscle and EDL muscle was analyzed before and after inducing fatigue (Figure 2). A similar effect could be observed in the work of García et al. [18] in mouse muscle fibers where in the presence of diazoxide the ROS production rate was lower compared to the untreated group. This effect was attributed to the opening of mitoKATP channels, which improves contraction and decreases fatigue, thus reducing ROS levels [18,41].

On the other hand, diazoxide can also decrease ROS levels in obesity by preventing mitochondrial dysfunction produced by hyperinsulinemia and hyperglycemia triggered by increased intramuscular fat [5,6,7]. This protective effect results from inhibiting insulin secretion and improving insulin sensitivity and glucose transport [18], which is also attributed to the characteristic of increasing antioxidant defenses, which favors the decrease of ROS levels [42,43]. In the same way, it was observed how exercise decreased ROS levels in groups of obese rats (Figure 2A,B). This effect of exercise was also observed in the study by Ji et al. [23], where a group of rats was subjected to an exercise regimen where decreased ROS levels were observed compared to the untrained group, in response to an adaptation in their antioxidant systems. This protective role of exercise in obesity could be attributed to the production and activity of antioxidant enzymes, in addition to the activation of numerous signaling cascades involved in exercise [7,23,24].

Similarly, exercise could decrease ROS levels during obesity by increasing the expression and participation of sarcolemmal KATP channels, improving muscle contraction and resistance to fatigue [13,34,38]. In the same way, these positive effects of exercise are attributed to the decrease in intramuscular fat due to the increase in skeletal muscle metabolism [5], or the optimization of signaling cascades related to insulin sensitivity, glucose transport, and inflammatory processes, which in turn decreases ROS production [7,44]. In this experiment series, the muscles of the groups subjected to the exercise protocol analyzed after fatigue showed the same ROS levels as the muscles analyzed before fatigue, an effect that was not observed in the rest of the groups, which can be explained with the concept of hormesis, which describes the response related to a beneficial stress factor at moderate levels and harmful at high levels [4].

It has been observed that one of the main effects of the increase in ROS is the damage to biomolecules [45], so in this study, the levels of TBARS were quantified as an indicator of lipid peroxidation and oxidative stress. This work observed that lipid peroxidation levels were increased in obese rats, both in the soleus and in EDL muscles analyzed before and after inducing fatigue (Figure 2C,D). However, the group of obese rats treated with diazoxide showed decreased lipid peroxidation levels in both muscles (Figure 2C,D). Farahini et al. [46] observed that pretreatment with diazoxide contributed to muscle tissue resistance against ischemic damage by decreasing lipid peroxidation. The same was appreciated by Moghtadaei et al. [42], where they observed this protective effect of diazoxide in skeletal muscle ischemia, as the levels of lipid peroxidation were decreased in the group preconditioned with diazoxide.

Similarly, the experimental series results showed decreased lipid peroxidation levels in the group of obese-exercise rats (Figure 2C,D). This effect with the implementation of an exercise protocol was previously observed by Lambertucci et al. [24], where the levels of lipid peroxidation of muscle tissue from trained rats were decreased. The decrease in both ROS levels and lipid peroxidation with diazoxide treatment and exercise, and the combination of both is directly proportional to muscle contraction improvement and increased resistance time to fatigue.

Cells have antioxidant systems that protect them against oxidative damage. This includes the antioxidant enzyme glutathione peroxidase, the synthesis of which can be modified by exercise, diet, and age [40]. Our results showed that both the levels of GSH + GSSG of the soleus muscle (Figure 3A) and EDL muscle (Figure 4A), as well as of GSH of the soleus muscle (Figure 3B) and EDL muscle (Figure 4B) were decreased during obesity, while the GSSG levels of the soleus muscle (Figure 3C) and EDL muscle (Figure 4C) were increased. The decrease in the content of GSH + GSSG suggests that obesity affects the synthesis of this antioxidant, possibly because ROS that are overproduced during this pathology are involved in activating various signaling pathways that negatively affect the expression of genes involved. The synthesis and content of GSH decreased [47], suggesting that obesity affects the antioxidant capacity. Diazoxide treatment, exercise, and a combination of both increased GSH + GSSG and GSH levels and, to a lesser extent, reduced GSSG levels in obesity. Moghtadaei et al. [42] reported that glutathione activity was increased in skeletal muscle treated with this drug during ischemia-reperfusion, concerning the muscle without treatment.

On the other hand, exercise presented this same protective effect during obesity since it enhances antioxidant activity [24]. Both the protective effect of diazoxide and exercise may be because these treatments regulate signaling pathways for the activation of genes that regulate the synthesis of antioxidant enzymes [42], in addition to the fact that glutathione synthesis is widely dependent on ATP concentrations. Both diazoxide treatment and exercise favor its production [36].

## 5. Conclusions

This is the first study, which shows how diazoxide and exercise and the combination of both improve the contraction and functioning of muscle fibers in obesity by reducing oxidative stress. In conclusion, diazoxide and exercise improves muscle contraction in obesity by decreasing ROS levels, lipid peroxidation, and improving the redox status of glutathione.

## Figures and Tables

**Table 1:** Experimental groups.

Groups	Diet	Diazoxide 35 mg/kg	Exercise Moderate Intensity
Control (C)	Standard rodent chow^®^	no	no
Diazoxide (D)	Standard rodent chow^®^	yes	no
Exercise (E)	Standard rodent chow^®^	no	yes
Obese (O)	High fat diet	no	no
Obese diazoxide (OD)	High fat diet	yes	no
Obese exercise (OE)	High fat diet	no	yes
Obese exercise diazoxide (OED)	High fat diet	yes	yes
Exercise diazoxide (ED)	Standard rodent chow^®^	yes	yes

**Table 2:** Exercise protocol. Exercise protocol per week for groups: E; exercise, ED; diazoxide exercise, OE; obese exercise, OED; obese diazoxide exercise. The speed is displayed in meters per minute and the time for which this speed was applied is shown in parentheses.

Groups	E, ED	OE, OED
Week 1	10 m/min (10 min)	10 m/min (10 min)
Week 2	10 m/min (10 min) 16 m/min (5 min)	10 m/min (10 min) 16 m/min (5 min)
Week 3	10 m/min (5 min) 16 m/min (5 min) 22 m/min (5 min)	10 m/min (5 min) 16 m/min (10 min)
Week 4	10 m/min (5 min) 16 m/min (5 min) 22 m/min (5 min)	10 m/min (5 min) 16 m/min (10 min)
Week 5	10 m/min (5 min) 16 m/min (5 min) 22 m/min (10 min)	10 m/min (5 min) 16 m/min (15 min)

**Table 3:** Weight and glucose at the end of the treatments; C: control; D: diazoxide; E: exercise; O: obese; OD: obese diazoxide; OE: obese exercise; OED: obese exercise diazoxide; ED: exercise diazoxide. The data are represented as the mean ± standard error. Different letters indicate statistically significant differences between groups (*p* < 0.05) one-way ANOVA, Tukey post-hoc test, *n* = 8.

Groups	Bodyweight (g)	Glucose
C	323.80 ± 2.80 g ^a^	77 ± 1.13 mg/dL ^a^
D	322.12 ± 2.27 g ^a^	85 ± 1.56 mg/dL ^b^
E	324.28 ± 5.37 g ^a^	70 ± 1.47 mg/dL ^c^
O	552.57 ± 3.61 g ^b^	98 ± 1.25 mg/dL ^d^
OD	451.16 ± 19.34 g ^c^	84 ± 1.65 mg/dL ^b^
OE	455.19 ± 21.66 g ^c^	77 ± 1.51 mg/dL ^a^
OED	427.16 ± 14.89 g ^c^	72 ± 1.84 mg/dL ^ac^
ED	354.33 ± 2.12 g ^a^	75 ± 2.80 mg/dL ^ac^
